# Evaluation of Four 3D Facial Scanning Technologies: From Photogrammetry to Structured-Light Systems in Clinical Dentistry

**DOI:** 10.3390/dj14020113

**Published:** 2026-02-14

**Authors:** Oana Elena Burlacu Vatamanu, Corina Marilena Cristache, Sergiu Drafta, Vanda Roxana Nimigean

**Affiliations:** 1 Doctoral School, “Carol Davila” University of Medicine and Pharmacy, 37 Dionisie Lupu Street, 020021 Bucharest, Romania; oana-elena.burlacu-vatamanu@drd.umfcd.ro; 2 Department of Dental Techniques, Faculty of Midwifery and Nursing, “Carol Davila” University of Medicine and Pharmacy, 8, Eroii Sanitari Blvd., 050474 Bucharest, Romania; 3 Department of Fixed Dental Prosthetics and Occlusion, Faculty of Dental Medicine, “Carol Davila” University of Medicine and Pharmacy, 19 Plevnei Ave., 010221 Bucharest, Romania; sergiu.drafta@umfcd.ro; 4 Department of Oral Rehabilitation, Faculty of Dentistry, “Carol Davila” University of Medicine and Pharmacy, 4-6 Eforie Street, 050037 Bucharest, Romania; vanda.nimigean@umfcd.ro

**Keywords:** 3D facial scanning, virtual dental patient, structured-light scanner, photogrammetry, neural radiance fields, digital dentistry, facial anthropometry

## Abstract

**Background/Objectives:** Accurate three-dimensional (3D) facial scanning is increasingly important in digital dentistry for diagnosis, treatment planning, and virtual patient creation. Multiple facial scanning technologies are available; however, their metric reliability varies depending on acquisition principles and anatomical orientation. This study aimed to evaluate the trueness, orientation-dependent performance (vertical midline versus horizontal facial measurements), and scanning time of four facial scanning technologies using calibrated manual anthropometry as the reference standard. **Methods:** Thirty dentate adult participants received adhesive fiducial markers on five predefined facial landmarks. Four linear facial distances were measured clinically using a digital caliper and compared with corresponding measurements obtained from standardized 3D facial scans. Digital measurements were extracted following uniform metric normalization. Inter-examiner reliability, measurement trueness, orientation-related differences, and scanning time were analyzed. **Results:** Inter-examiner reliability was excellent for both clinical and digital measurements (ICC > 0.93). All facial scanning technologies significantly overestimated manual distances (*p* < 0.001). The structured-light scanning system showed the smallest deviations (typically <1 mm) and the highest overall accuracy, followed by the depth-fusion system, while photogrammetry-based and NeRF-based approaches demonstrated larger errors, frequently exceeding 2–3 mm. Horizontal facial distances consistently showed greater deviations than vertical midline measurements across all systems. Scanning time differed significantly between technologies, with passive image-based approaches being the fastest and NeRF-based acquisition requiring the longest capture time. **Conclusions:** Active structured-light facial scanning demonstrated the highest trueness for linear facial anthropometry, whereas passive photogrammetry and NeRF-based approaches showed lower metric trueness and are currently more suitable for educational applications.

## 1. Introduction

The human face has always played a central role in dental diagnosis and treatment planning, as facial proportions, symmetry, and soft-tissue dynamics directly influence aesthetic and functional rehabilitation. Early morphological research established the foundations for quantitative facial analysis, and evolving digital technologies have now made facial characterization accessible beyond specialized research environments [1,2]. Over the last decade, three-dimensional (3D) facial scanning has rapidly transitioned from experimental use to practical clinical applications, supporting diagnostic protocols, treatment simulation [3], and patient communication in dentistry.

The integration of 3D facial scans with intraoral optical impressions and cone-beam computed tomography (CBCT) data enables the construction of a comprehensive virtual dental patient [4,5]. This combined digital representation supports facially driven planning by linking soft-tissue morphology with occlusion and skeletal structures in a unified environment [6,7,8,9]. Consequently, the metric reliability of facial scans is clinically relevant for applications in orthodontics, implant-prosthodontics, maxillofacial rehabilitation, and orthognathic planning [10].

A broad spectrum of acquisition principles is now available for 3D facial capture. High-precision clinical systems typically employ structured-light projection or stereophotogrammetry, producing accurate and textured facial surfaces suitable for static or dynamic evaluation [11,12]. Other systems generate facial geometry through multi-view photogrammetry, either via controlled head positioning or through mobile applications that reconstruct 3D surfaces from image sequences. Recent advances have introduced artificial intelligence (AI)–based reconstruction using neural radiance field (NeRF) models, which estimate 3D facial structure from standard video recordings, superseding the conventional triangulation-based approach [12,13]. These innovations reflect a shift toward accessible and portable tools capable of supporting digital workflows and remote data acquisition, particularly for cases requiring longitudinal monitoring or multidisciplinary collaboration [8,14].

Despite growing adoption, key practical limitations remain. Active scanners often require controlled environments and trained operators, while smartphone-based and AI-driven systems improve accessibility at the cost of reduced trueness and repeatability in clinically relevant regions [10,13,14]. Notably, few standardized comparative studies have assessed contemporary scanning systems under identical conditions using clinically meaningful reference standards. Consequently, it is unclear which acquisition modality offers the best balance of accuracy, acquisition time, and workflow feasibility for dental applications.

To address this knowledge gap, the present study evaluates four accessible facial scanning solutions—representing structured-light, stereophotogrammetric, and NeRF-based platforms—and compares their performance against manual anthropometry across multiple soft-tissue distances. Specifically, the study objectives are: (1) to compare the trueness of the four facial scanning technologies against manual anthropometric measurements; (2) to assess the influence of anatomical orientation on measurement variability; and (3) to evaluate acquisition time as a parameter of workflow feasibility in clinical dentistry. The null hypothesis stated that no statistically significant differences would be found among the four scanning systems in terms of measurement trueness, orientation-dependent performance, or acquisition time relative to manual anthropometry.

## 2. Materials and Methods

### 2.1. Study Design

This investigation was designed as a method comparison and reproducibility study, aiming to evaluate the agreement between clinical (manual) facial measurements and corresponding measurements derived from multiple 3D scanning systems. Additionally, the study assessed the inter-examiner reliability of both clinical and digital measurement methods performed by two independent examiners.

### 2.2. Ethical Approval and Participant Selection

This prospective study was conducted at a clinical facility affiliated with the “Carol Davila” University of Medicine and Pharmacy in Bucharest, Romania, where participant recruitment and data acquisition took place. The study adhered to the ethical standards outlined in the World Medical Association’s Declaration of Helsinki and complied with applicable institutional and national ethical regulations. Approval was granted by the Ethics Committee of the “Carol Davila” University of Medicine and Pharmacy (approval number 36368/2023), and written informed consent was obtained from all adult participants.

Participants were selected from a cohort of third-year undergraduate students enrolled in the Dental Technique program. Inclusion criteria comprised the following:

- Absence of craniofacial or dental anomalies;
- Complete dentition without edentulous spaces;
- No history of facial trauma or maxillofacial surgery;
- No clinically evident facial asymmetry.

The inclusion of young adult participants without clinically evident facial asymmetry was intentional and aimed at minimizing confounding variables related to age-related soft-tissue changes, such as skin laxity, ptosis, and wrinkle formation. This controlled sample allowed the present study to primarily assess scanner-related measurement trueness and orientation-dependent errors, rather than biological variability.

### 2.3. Sample Size Calculation

An a priori power analysis was performed using G*Power (version 3.1) to determine the minimum sample size required to assess the association between physical reference measurements and those obtained from four different facial scanners. The calculation was based on an anticipated effect size of |ρ| = 0.65, derived from comparable studies [15,16,17], using a two-tailed bivariate correlation model. Although agreement and absolute measurement errors represented important inferential outcomes of the present study, validated a priori power calculation strategies for multi-scanner agreement designs are not well-established. Therefore, a correlation-based model was selected as a pragmatic proxy to ensure adequate linear association between manual and digital measurements. This approach has been applied in comparable facial scanning studies [15,16,17]. To control for multiple comparisons across the four scanners, the significance level was adjusted using a Bonferroni correction (α = 0.0125), with a desired statistical power (1 − β) of 0.90. The analysis indicated that a minimum of 27 participants would be required to detect a correlation of this magnitude. To further enhance statistical robustness and accommodate potential data loss, a total of 30 adult participants were enrolled in the study. Post hoc precision analyses indicated that the achieved sample (*n* = 30) provided >0.90 observed power for all primary paired comparisons at α = 0.001, confirming adequate sensitivity for detecting systematic differences between manual and digital measurements.

### 2.4. Data Acquisition

#### 2.4.1. Clinical Measurements (Reference Standard)

Adhesive fiducial markers (diameter: 10 mm; white core: 6 mm; black border: 2 mm on each side) were placed on predefined soft-tissue anatomical sites. Linear distances between the center points of these markers were obtained using a calibrated anthropometric digital caliper (precision: 0.01 mm).

Manual caliper measurements using adhesive fiducial markers were employed as a clinical reference standard instead of true anatomical anthropometry. While this approach reflects common clinical practice for facial measurements, it may introduce minor sources of bias related to soft-tissue compression, marker thickness, and local alteration of surface geometry. These effects were minimized through standardized marker placement and gentle caliper contact; however, they cannot be completely eliminated in an in vivo setting.

All measurements were performed independently by two examiners (O.E.B.V. and C.M.C.). Each examiner recorded three consecutive readings for each landmark pair, and the mean value of the three measurements served as the reference clinical value.

The same adhesive markers remained fixed on the participant’s face during clinical measurements and throughout all 3D scanning procedures to ensure spatial consistency.

#### 2.4.2. 3D Scanning Procedures

Four digital facial acquisition systems were used, representing different 3D reconstruction technologies:

Revopoint POP 2 (Revopoint 3D Technologies Inc., Shenzhen, China) is a structured-light handheld scanner that projects patterned infrared light onto the facial surface and reconstructs 3D geometry through binocular depth analysis. For complete geometry acquisition, the operator moves around the participant during scanning [10].

Bellus 3D Face Camera Pro (Bellus3D Inc., Campbell, CA, USA) is an infrared depth-sensing camera that combines structured-light projection with front-facing camera depth fusion. Unlike handheld systems, Bellus 3D acquisition relies on guided head movements performed by the participant, generating a standardized capture pathway and a predictable scan duration [18,19].

Polycam (Polycam Inc., San Francisco, CA, USA) is a smartphone-based photogrammetry application that reconstructs 3D facial geometry from multiple high-resolution 2D images acquired around the subject. This approach requires minimal operator expertise but is highly influenced by controlled lighting and sufficient image overlap [20,21]. Photographic datasets for the photogrammetry-based digital models were acquired using an iPhone 13 Pro (Apple Inc., Cupertino, CA, USA). Each participant remained seated upright on a high-back chair while the operator performed a 360° circular sweep at a constant distance of approximately 70 cm from the face. Technical parameters included: 12-megapixel Portrait Mode, 26 mm wide lens, ISO capped at 400, Exposure compensation of +0.7 EV.

LumaAI (Luma Labs Inc., Palo Alto, CA, USA) is an AI-driven scanning platform that employs Neural Radiance Fields (NeRF) for multi-view 3D reconstruction. The scan consists of image sequences captured from three 360° rotational levels, significantly increasing scan duration while enabling dense geometric acquisition and high-fidelity rendering [21,22]. The same iPhone model was used for both Polycam and LumaAI captures.

Polycam and LumaAI scans were recorded under identical illumination conditions using two Haiser Softbox units (6500 K, 1200 lumens), positioned at 45° relative to the facial midline. Quality control criteria included monitoring RGB histograms, ensuring ≥60% frame overlap, and verifying sharp focus on critical anatomical regions (pupils, nasal tip, ear lobe).

For Revopoint POP 2, Polycam and LumaAI, participants remained motionless while the operator circled the head with the device. For Bellus3D, participants followed guided head movements displayed by the application using the device’s front-facing camera.

**Figure 1:**
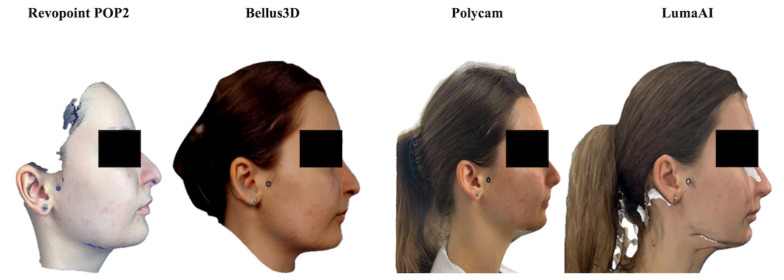
Facial scans acquired using the four different devices, with adhesive fiducial markers applied at corresponding soft-tissue anatomical sites. Differences in lateral boundary extent reflect device-specific fields of view and reconstruction strategies. All anatomical regions relevant to the measurement protocol were consistently captured across systems.

Each scan was exported in OBJ. format for subsequent analysis in Blender 4.4.0 (Blender Foundation, Amsterdam, The Netherlands) (Figure 1).

Scanning time was operationally defined as the active acquisition time only, measured from the start of facial data capture to the completion of the scanning procedure. Post-acquisition processing time, including local or cloud-based reconstruction, was not included, as the present analysis focused on operator-dependent acquisition efficiency. This definition was applied consistently across all systems to ensure fair comparison of scanning performance.

### 2.5. Facial Landmarks and Digital Annotations

**Figure 2:**
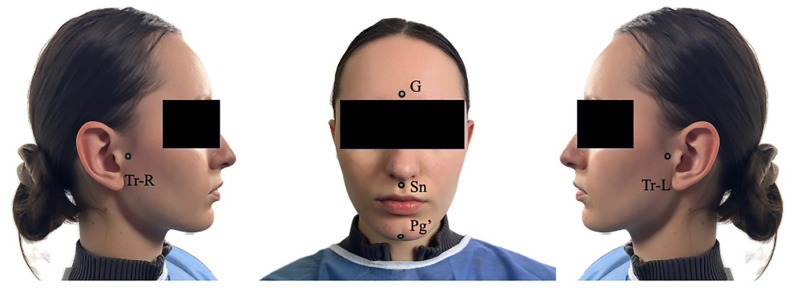
Landmark positions used in the measurement protocol: Glabella (G), Subnasale (Sn), Soft Pogonion (Pg′), Right Tragus (Tr-R), and Left Tragus (Tr-L).

The adhesive fiducial markers were applied at the following soft-tissue anatomical sites (Figure 2):

- Glabella (G)
- Subnasale (Sn)
- Soft Pogonion (Pg′)
- Right Tragus (Tr-R)
- Left Tragus (Tr-L)

For digital models, equivalent points were recreated as virtual Empty objects placed manually in Blender using orthographic frontal and lateral views. Multi-angle inspection was used to ensure precise alignment and reproducibility between models.

Prior to data extraction, examiners were trained using pilot facial models to ensure consistent identification of the selected landmarks. During digital landmark placement, examiners were blinded to the corresponding clinical caliper measurements.

### 2.6. Measurement Protocol

**Figure 3:**
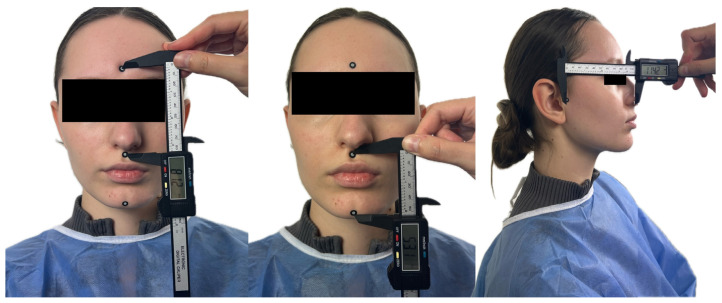
Linear anthropometric measurements obtained using a digital caliper, performed between the centers of fiducial markers applied to corresponding soft-tissue anatomical sites.

Four linear Euclidean distances were measured (Figure 3):

- D1: Glabella–Subnasale
- D2: Subnasale–Soft Pogonion
- D3: Subnasale–Left Tragus
- D4: Subnasale–Right Tragus

Distances were expressed in millimeters (mm) and extracted using vertex-to-vertex coordinate differences for digital models and anthropometric caliper readings for clinical measurements. The digital value recorded by each examiner for each scanner was compared with the corresponding mean clinical value obtained by the same examiner.

### 2.7. Digital Workflow and Scaling

All 3D models were imported into Blender 4.4.0 for measurements

Structured-light system models served as the reference geometry due to their detailed anatomical fidelity.

All 3D facial models were brought to the same real-world metric scale using a custom Python 3.10 script implemented in Blender.

**Figure 4:**
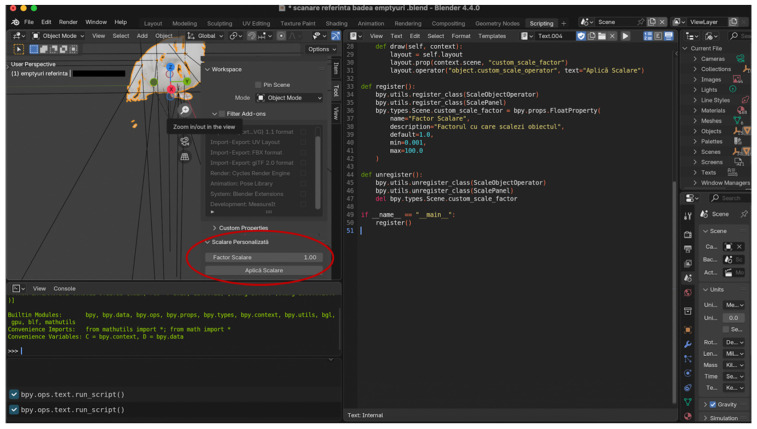
Blender interface illustrating the custom Python script used to perform uniform scaling across all 3D scans. The circled button represents a control parameter created in Blender via Python to ensure each newly imported scan is automatically scaled in a consistent and reproducible manner.

A single stable vertical midline facial dimension was used exclusively to calculate a global scaling factor, which was then uniformly applied to the entire model (X, Y, and Z axes) (Figure 4).

Neither vertical nor horizontal dimensions were selectively modified, corrected, or calibrated. The Python-based scaling procedure served only to ensure dimensional consistency across datasets and did not alter the intrinsic geometric relationships produced by each scanning system.

No axis-specific deformation, selective rescaling, or post-acquisition geometric correction was performed.

**Figure 5:**
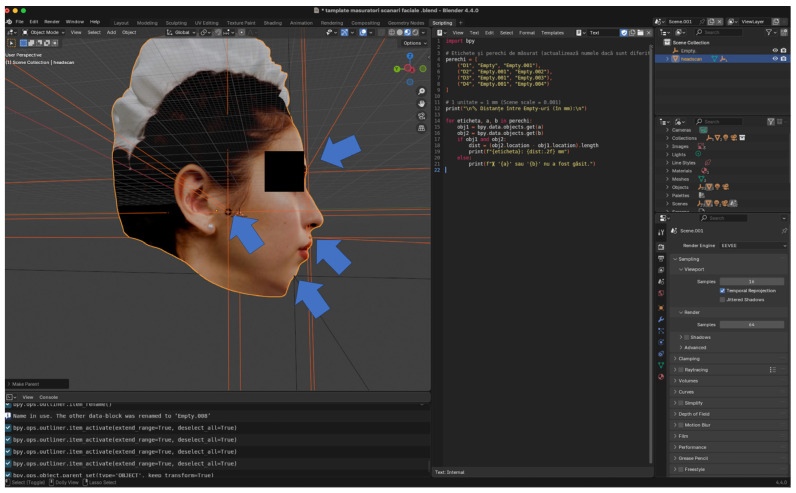
3D facial landmark placement and automated measurement extraction using Python in Blender. The arrows indicate the fiducial markers used for the measurements.

Distance measurements were computed using a second integrated Python script, which extracted Euclidean distances between selected Empty objects (Figure 5). No smoothing, remeshing, or mesh modification was performed to preserve native scan geometry.

### 2.8. Statistical Analysis

Statistical analyses were performed in the open-source statistical software package JASP (version 0.95.4). Normality was assessed with the Shapiro–Wilk test. Paired-samples t-tests and Wilcoxon signed-rank tests were used to compare manual and digital measurements. Differences in scanning time were evaluated with repeated-measures ANOVA and Bonferroni post hoc tests. Global accuracy among scanners was assessed using the Friedman test with Conover post hoc comparisons. Vertical versus horizontal differences were analyzed using paired t-tests. Statistical significance was set at *p* < 0.001. While the a priori power analysis employed a Bonferroni-adjusted α = 0.0125 for correlation estimates, a more conservative threshold of α = 0.001 was applied to the paired difference tests to control Type I error across multiple scanner × distance comparisons. This stricter α-level was chosen to reduce the risk of false positives given the number of repeated measures.

## 3. Results

Thirty dentate participants, ranging in age from 20 to 27 years, were enrolled. The mean age of the sample was 21.57 years (SD 1.38).

The mean intraclass correlation coefficient (ICC) between the two investigators exceeded 0.93 for both clinical and digital linear measurements, indicating excellent inter-examiner reliability.

Table 1 summarizes the paired comparisons between manual measurements and the corresponding digital measurements obtained from the four facial scanning systems across the four linear distances evaluated. Mean differences represent signed values (Manual − Digital), with negative values indicating systematic overestimation by digital measurements. Cohen’s dᶻ represents the paired-sample effect size calculated using the standard deviation of paired differences. Large d^z^ values reflect high consistency of paired differences rather than large absolute measurement errors.

All scanners produced significantly larger digital values compared with the manual reference (all *p* < 0.001), although the magnitude of deviation varied among devices and distances. Revopoint POP 2 showed the smallest overall differences, with mean deviations ranging from −0.04 mm to −0.99 mm (Cohen’s d = 0.31–1.70). Bellus 3D demonstrated moderate discrepancies, particularly pronounced for D3 (mean difference −1.90 mm; d = 4.06).

Significantly larger deviations were observed for Polycam and LumaAI, with mean differences reaching up to −2.87 mm and −3.29 mm, respectively, and very large effect sizes (d up to 5.18).

Although digital measurements consistently overestimated manual values, the pattern of deviation differed across devices. Revopoint demonstrated the most consistent performance, whereas Polycam and LumaAI exhibited the largest numerical deviations. Bellus presented minimal differences for D1, D2, and D4, but a marked overestimation for D3.

Shapiro–Wilk testing indicated non-normality for several paired differences, but non-parametric (Wilcoxon) tests confirmed the same significant direction of effects.

A repeated-measures ANOVA (Table 2) revealed a highly significant effect of scanner type on scanning time (Greenhouse–Geisser corrected: F(1.49, 43.25) = 2851, *p* < 0.001). Post hoc pairwise comparisons (Table 2) showed that the fastest acquisition was obtained with Polycam, which required significantly less time than both Revopoint POP 2 (mean difference = −28.09 s, *p* < 0.001) and Bellus 3D (mean difference = −12.50 s, *p* < 0.001).

Bellus 3D also performed significantly faster than Revopoint (mean difference = −15.59 s, *p* < 0.001).

In contrast, LumaAI demonstrated the longest scanning time, being significantly slower than all other devices by margins exceeding 96–124 s (all *p* < 0.001).

The Friedman test (Table 3) revealed a highly significant effect of scanner type on measurement error (χ^2^(3) = 85.48, *p* < 0.001), with a very large effect size (Kendall’s W = 0.950). Mean rank values indicated clear differences in accuracy among devices, with Revopoint POP 2 showing the smallest overall error (mean rank = 30.0), followed by Bellus 3D (61.0), Polycam (92.0) and LumaAI (117.0).

Conover post hoc comparisons confirmed that all scanners differed significantly from each other (all *p* < 0.001, Bonferroni-adjusted). These results demonstrate that Revopoint achieved the highest overall accuracy relative to manual anthropometric measurements, while Luma AI exhibited the largest deviations.

Paired-samples t-tests (Table 4) revealed significantly larger errors for the horizontal distances (D3–D4) compared with the vertical distances (D1–D2) for all four scanners (all *p* < 0.001).

For Revopoint, horizontal errors (mean = 0.876 mm) were substantially greater than vertical errors (mean = 0.040 mm), t(29) = −11.56, *p* < 0.001.

Bellus 3D showed a similar pattern, with horizontal deviations (mean = 2.136 mm) significantly exceeding vertical ones (mean = 0.298 mm), t(29) = −9.93, *p* < 0.001.

Polycam exhibited higher errors on horizontal measurements (mean = 2.688 mm vs. re1.297 mm), t(29) = −7.33, *p* < 0.001.

LumaAI demonstrated the largest orientation-related discrepancy (horizontal = 3.095 mm vs. vertical = 1.587 mm), t(29) = −6.34, *p* < 0.001.

## 4. Discussion

This investigation provides a comprehensive in vivo comparison of four widely available facial scanning systems—Revopoint POP 2, Bellus 3D Face Camera Pro, Polycam, and LumaAI—using calibrated linear anthropometry as the reference standard. Unlike most previous investigations, which typically evaluate a single scanning technology or similar device class [23], this study simultaneously assessed four different 3D reconstruction philosophies: handheld structured light, guided depth fusion, smartphone photogrammetry, and AI-driven NeRF reconstruction. All scanners were tested using the same subjects, fiducial landmark protocol, controlled illumination, and repeated measurements by two calibrated examiners, allowing for a meaningful comparison based on both accuracy and workflow performance. These methodological features differentiate this study from those conducted under controlled in vitro conditions [24,25] or with homogeneous device designs.

### 4.1. Inter-Examiner Reliability

Excellent inter-examiner reliability was demonstrated, with ICC values above 0.93 for both manual and digital measurements. This high reproducibility is consistent with previous research showing that when landmark definitions are standardized and operators are familiar with digital landmarking protocols, facial scanner reproducibility approaches unity [17]. These findings indicate that examiner-related variability was minimized in this study, allowing system-related measurement behavior to be evaluated independently.

### 4.2. Agreement with Manual Anthropometry

The present study prioritized paired difference testing and ICC-based reliability metrics, as these approaches more directly assess agreement and measurement bias than correlation coefficients alone. Although a correlation-based model was used for the initial power estimation—reflecting the absence of standardized methods for sample size determination in multi-scanner agreement studies—post hoc power analyses confirmed that the final sample (*n* = 30) maintained sufficient sensitivity to detect systematic paired differences at α = 0.001. These results support the statistical robustness of the findings and confirm that the sample size was adequate for the study’s primary outcomes.

All four digital acquisition systems demonstrated statistically significant overestimation of linear distances compared with manual measurements. This systematic inflation has been reported in previous optical scanning research, including the in vitro validation by Amornvit and Sanohkan, who observed similar overestimation relative to caliper measurements, especially as distance increased [8]. Comparable proportional bias has been described by Puleio et al., who validated the Planmeca ProFace system and noted that digital values exceeded caliper measurements to a greater extent at larger spans [26]. This pattern aligns with the present findings, in which horizontal tragus–subnasale distances exhibited greater inflation than midline vertical measurements, indicating that reconstruction error scales with anatomical trajectory length. Furthermore, Stancioiu et al. demonstrated that structured-light MetiSmile scans can achieve sub-millimetric deviations and high repeatability for orthodontic parameters [7], reinforcing the capacity of active depth-sensing systems to approximate manual anthropometry with clinically acceptable precision.

These deviations likely arise from smoothing, meshing interpolation, and the optical challenges of reconstructing soft-tissue contours.

Device-specific variation was substantial. Revopoint POP 2 showed the smallest and most consistent deviations—frequently below 1 mm—whereas Bellus3D demonstrated acceptable performance for midline distances but displayed a pronounced overestimation for horizontal tragus–subnasale measurements. Polycam and LumaAI produced the largest numerical discrepancies, exceeding 2–3 mm in several cases. This hierarchy is congruent with the technical properties of each scanner and corresponds well with the in vitro literature, in which structured-light scanners consistently achieve the highest accuracy and image-based approaches the lowest [27].

### 4.3. Active Versus Passive Acquisition Methodologies

Differences in accuracy can be attributed to the acquisition philosophy. Revopoint POP 2 and Bellus3D are active systems, projecting structured or infrared light to triangulate depth and generate uniform point clouds. Active depth sensing is inherently more robust to texture variation, shadowing, and complex curvature, which explains the higher trueness achieved by Revopoint and the moderate performance of Bellus3D. The previously published research confirms that structured-light systems consistently outperform passive smartphone photogrammetry in terms of metric fidelity, particularly when scanning soft tissues with pronounced curvature or reduced reflectance [28]. However, differences in observed trueness reflect not only acquisition physics but also the interaction between optical limits, anatomical complexity, and the reference methodology used for validation.

Polycam and LumaAI represent passive reconstruction frameworks, although using different mathematical approaches—traditional photogrammetry versus neural radiance fields (NeRF). Passive reconstruction infers geometry from multiple 2D images without direct depth projection, making it highly sensitive to lighting, image overlap, motion, and camera stability. Even under optimized environmental conditions, passive scanners struggle with lateral concavities, hair regions, and curved soft-tissue contours, explaining the larger deviations observed in this study [28].

LumaAI additionally shows that high visual realism does not guarantee metric fidelity; neural field estimation produces compelling textures but may introduce undetectable geometric distortion. In the present study, the NeRF-based system (LumaAI) exhibited the largest deviations relative to the clinical reference, particularly for horizontal facial dimensions, despite producing visually realistic surface renderings. This finding underscores that high photorealism does not equate to metric fidelity. While NeRF-based reconstruction offers compelling visualization, the absence of explicit depth calibration limits its current suitability for quantitative facial anthropometry [21].

### 4.4. Orientation-Dependent Accuracy

A consistent and clinically relevant finding was the disparity between vertical and horizontal distances. All scanners displayed significantly higher errors for D3 and D4 (tragus–subnasale) compared with D1 and D2 (midline vertical distances). Horizontal measurements traverse regions of greater curvature, soft-tissue mobility, and shadowing, increasing the risk of interpolation error. Similar directional discrepancies have been reported in optical scanning literature, where lateral facial surfaces and recessed regions—such as commissures and auricular zones—produce lower depth mapping accuracy [28].

These results confirm that facial scanning accuracy is not isotropic, and clinicians should interpret lateral dimensions with greater caution. This orientation-dependent pattern should also be interpreted in light of the global scaling procedure applied for metric normalization, which ensured dimensional consistency across models but did not correct orientation-specific geometric distortions.

### 4.5. Workflow Characteristics and Scanning Time

Scanning time emerged as a key differentiating factor. Polycam exhibited the fastest acquisition, followed by Bellus3D and Revopoint POP 2. LumaAI required substantially longer scanning due to its multi-sequence NeRF capture methodology. Although Amornvit and Sanohkan found that smartphone scanning was fastest and structured light slowest in vitro [8], our in vivo data extend this observation by demonstrating that speed is inversely correlated with metric accuracy. Fast smartphone scanning offers clinical convenience and visualization benefits but sacrifices depth fidelity. Conversely, structured-light acquisition requires longer scanning but provides superior quantitative performance. This reinforces the principle that device choice should depend on the clinical objective rather than the convenience of capture.

The performance hierarchy observed in this study—structured light > depth fusion > photogrammetry > NeRF—is consistent with published evidence on extraoral and intraoral scanners. Structured-light devices frequently achieve submillimetric trueness when anatomical surfaces are stable and measurement protocols are standardized. Studies on intraoral scanners show that trueness deteriorates with span length, curvature, and scanning extent, supporting the observation that reconstruction error accumulates along more complex surfaces. Similar conclusions were reached by Mangano et al. [29], Braian et al. [30], Abduo et al. [31], and Medina-Sotomayor et al. [32], who consistently report that larger scan spans and complex anatomy reduce metric reliability. Additionally, the in vitro study demonstrated that no scanner except EinScan Pro 2X Plus could reliably capture narrow depth features, supporting the present in vivo finding that lateral anatomy remains more error-prone [8,28].

### 4.6. Clinical Implications

In dental and prosthodontic applications, active structured-light scanning remains the preferred modality for tasks requiring precise anthropometric evaluation, including virtual facebow transfers, symmetry analysis, and planning of implant-supported prostheses. Bellus3D may be appropriate for esthetic visualization and digital smile design, but lateral anthropometry should be interpreted with caution. Unfortunately, Bellus3D is no longer commercially available, as the company officially announced its decision to wind down operations by the end of 2022 [33]. Polycam and LumaAI are best suited to visualization, teledentistry, patient communication, or educational simulations, and should not be used as stand-alone tools for quantitative anthropometry unless supplemented with calibrated scaling, optimized redundancy, or hybrid depth sensing.

Regulatory and ethical considerations further affect mobile acquisition. As noted in the recent literature, Medical Device Regulation (MDR)-compliant dental apps have become scarce, and handling facial biometric information requires robust data protection and transparency. Clinicians should ensure that scanning applications meet General Data Protection Regulation (GDPR)/MDR requirements, with appropriate informed consent regarding data storage and cloud processing [34].

### 4.7. Limitations and Future Directions

This study has several limitations that should be acknowledged. Only four linear facial distances were analyzed, and digital landmark placement was examiner-dependent, which may introduce a degree of operator-related variability. In addition, all scans were acquired under controlled conditions and included only young adult participants with symmetrical facial anatomy. Different age groups, age-related soft-tissue changes (such as ptosis and loss of skin tone), facial asymmetries, edentulous anatomy, or intraoperative movement and involuntary patient movements may influence scanning trueness, particularly in an in vivo setting.

Facial scanning accuracy may also be affected by subtle motion artifacts, especially for acquisition protocols requiring longer capture times and for passive image-based systems. Furthermore, operator skill represents an additional limitation, particularly for handheld scanners that rely on manual control of scanning trajectory and distance. In the present study, all operators were equally trained and calibrated prior to data acquisition and followed standardized scanning protocols; however, operator-dependent variability cannot be completely eliminated and may be more pronounced in routine clinical practice.

Future research should integrate larger surface-based error analyses, multi-operator evaluations, and real-world clinical settings, as well as investigate progressive calibration and optimization strategies—particularly for passive and hybrid AI-based scanning modalities—to improve their suitability for quantitative facial anthropometry.

## 5. Conclusions

Within the limits of the present study, including evaluation of a restricted set of linear facial distances and testing under a controlled scanning environment, the following conclusions can be drawn:

- Active structured-light facial scanning demonstrated the highest trueness and reproducibility for linear facial anthropometric measurements.
- Depth-fusion facial scanning systems showed moderate accuracy, particularly for vertical midline dimensions, but exhibited increased errors for horizontal measurements.
- Passive smartphone photogrammetry and NeRF-based facial reconstruction presented significantly larger deviations, especially for lateral facial dimensions.
- Measurement accuracy was strongly influenced by acquisition technology, anatomical orientation, and workflow characteristics.
- Based on the present findings, active depth-sensing technologies are currently more suitable for clinical applications requiring precise facial anthropometry, while passive image-based systems are better suited for visualization, education, or telemedicine unless supplemented by robust metric calibration.

## Figures and Tables

**Table 1:** Comparison of clinical and digital linear measurements for each scanner (D1–D4).

Scanner	Distance	Manual Mean ± SD	Digital Mean ± SD	Mean Diff (M–D)	t	df	*p*	Cohen’s d^z^ (Paired)
Structured-light system (Revopoint)	D1	67.19 ± 2.77	67.23 ± 2.79	−0.04	−3.820	29	<0.001	−0.697
	D2	45.80 ± 2.48	45.84 ± 2.47	−0.04	−4.032	29	<0.001	−0.736
	D3	107.38 ± 4.63	108.13 ± 4.67	−0.75	−9.331	29	<0.001	−1.704
	D4	106.38 ± 5.16	107.37 ± 5.33	−0.99	−7.943	29	<0.001	−1.452
Depth-fusion system (Bellus 3D)	D1	67.48 ± 2.74	68.10 ± 2.49	−0.62	−3.350	29	0.002	−0.612
	D2	45.84 ± 2.48	46.10 ± 2.49	−0.26	−3.088	29	0.004	−0.563
	D3	107.47 ± 4.61	109.37 ± 4.77	−1.90	−22.295	29	<0.001	−4.061
	D4	106.41 ± 5.14	108.66 ± 5.10	−2.25	−2.715	29	0.011	−0.484
Photogrammetry-based system (Polycam)	D1	67.19 ± 2.77	68.54 ± 2.85	−1.35	−14.157	29	<0.001	−2.574
	D2	45.80 ± 2.48	47.04 ± 2.68	−1.24	−12.454	29	<0.001	−2.274
	D3	107.38 ± 4.63	109.88 ± 4.77	−2.50	−22.351	29	<0.001	−4.095
	D4	106.38 ± 5.16	109.25 ± 4.96	−2.87	−8.764	29	<0.001	−1.600
NeRF-based system (LumaAI)	D1	67.19 ± 2.78	68.61 ± 2.85	−1.42	−5.140	29	<0.001	−0.938
	D2	45.89 ± 2.50	47.55 ± 2.63	−1.66	−15.692	29	<0.001	−2.865
	D3	107.50 ± 4.58	110.25 ± 4.69	−2.75	−28.371	29	<0.001	−5.180
	D4	106.40 ± 5.24	109.69 ± 5.01	−3.29	−9.963	29	<0.001	−1.819

**Table 2:** Comparison of measurement time for clinical and digital linear assessments across all scanners (D1–D4).

Comparison	Mean Difference (s)	SE	df	t	Cohen’s d	*p* (Tukey)	*p* (Bonferroni)
Structured-light system vs. Depth-fusion system	+15.59	0.922	29	16.90	2.585	<0.001	<0.001
Structured-light system vs. Photogrammetry-based system	+28.09	0.922	29	30.48	4.658	<0.001	<0.001
Structured-light system vs. NeRF-based system	−96.22	2.031	29	−47.38	−15.958	<0.001	<0.001
Depth-fusion system vs. Photogrammetry-based system	+12.50	0.554	29	22.57	2.073	<0.001	<0.001
Depth-fusion system vs. NeRF-based system	−111.80	1.880	29	−59.48	−18.543	<0.001	<0.001
Photogrammetry-based system vs. NeRF-based system	−124.30	1.950	29	−63.75	−20.616	<0.001	<0.001

**Table 3:** Conover post hoc comparisons of global measurement accuracy across scanners (Friedman test).

Comparison	T-Stat	df	*p* (Bonferroni)	Interpretation
Structured-light system vs. Depth-fusion system	13.60	87	<0.001	Structured-light system more accurate
Structured-light system vs. Photogrammetry-based system	27.20	87	<0.001	Structured-light system more accurate
Structured-light system vs. NeRF-based system	38.17	87	<0.001	Structured-light system more accurate
Depth-fusion system vs. Photogrammetry-based system	13.60	87	<0.001	Depth-fusion system more accurate
Depth-fusion system vs. NeRF-based system	24.57	87	<0.001	Depth-fusion system more accurate
Photogrammetry-based system vs. NeRF-based system	10.97	87	<0.001	Photogrammetry-based system more accurate

**Table 4:** Comparison of vertical and horizontal measurement errors across the four scanners.

Scanner	Orientation	N	Mean (mm)	SD (mm)	SE (mm)
Structured-light system	Vertical	30	0.040	0.043	0.008
	Horizontal	30	0.876	0.386	0.070
Depth-fusion system	Vertical	30	0.298	0.361	0.066
	Horizontal	30	2.136	0.950	0.173
Photogrammetry-based system	Vertical	30	1.297	0.351	0.064
	Horizontal	30	2.688	0.957	0.175
NeRF-based system	Vertical	30	1.587	0.783	0.143
	Horizontal	30	3.095	0.958	0.175

## Data Availability

The original contributions presented in this study are included in the article. Further inquiries can be directed to the corresponding author.

## Abbreviations

The following abbreviations are used in this manuscript:

CBCT	Cone-beam computed tomography
AI	Artificial intelligence
NeRF	Neural radiance field
ICC	Intraclass correlation coefficient
MDR	Medical Device Regulation
GDPR	General Data Protection Regulation
